# Molecular Network-Based Drug Prediction in Thyroid Cancer

**DOI:** 10.3390/ijms20020263

**Published:** 2019-01-11

**Authors:** Xingyu Xu, Haixia Long, Baohang Xi, Binbin Ji, Zejun Li, Yunyue Dang, Caiying Jiang, Yuhua Yao, Jialiang Yang

**Affiliations:** 1 College of Life Sciences, Zhejiang Sci-Tech University, Hangzhou 310018, China; xingyuxu821@163.com (X.X.); xibaohang@sina.com (B.X.); jcy@zstu.edu.cn (C.J.); 2 School of Information Science and Technology, Hainan Normal University, Haikou 571158, China; myresearch_hainnu@163.com; 3 School of Mathematics and Statistics, Hainan Normal University, Haikou 570100, China; jibinbin1222@163.com (B.J.); d130937701380@163.com (Y.D.); 4 College of Information Science and Engineering, Hunan University, Changsha 410082, China; lzjfox@163.com

**Keywords:** thyroid cancer, drug redirection, differential gene, differential co-expression, co-expression network, gene perturbation

## Abstract

As a common malignant tumor disease, thyroid cancer lacks effective preventive and therapeutic drugs. Thus, it is crucial to provide an effective drug selection method for thyroid cancer patients. The connectivity map (CMAP) project provides an experimental validated strategy to repurpose and optimize cancer drugs, the rationale behind which is to select drugs to reverse the gene expression variations induced by cancer. However, it has a few limitations. Firstly, CMAP was performed on cell lines, which are usually different from human tissues. Secondly, only gene expression information was considered, while the information about gene regulations and modules/pathways was more or less ignored. In this study, we first measured comprehensively the perturbations of thyroid cancer on a patient including variations at gene expression level, gene co-expression level and gene module level. After that, we provided a drug selection pipeline to reverse the perturbations based on drug signatures derived from tissue studies. We applied the analyses pipeline to the cancer genome atlas (TCGA) thyroid cancer data consisting of 56 normal and 500 cancer samples. As a result, we obtained 812 up-regulated and 213 down-regulated genes, whose functions are significantly enriched in extracellular matrix and receptor localization to synapses. In addition, a total of 33,778 significant differentiated co-expressed gene pairs were found, which form a larger module associated with impaired immune function and low immunity. Finally, we predicted drugs and gene perturbations that could reverse the gene expression and co-expression changes incurred by the development of thyroid cancer through the Fisher’s exact test. Top predicted drugs included validated drugs like baclofen, nevirapine, glucocorticoid, formaldehyde and so on. Combining our analyses with literature mining, we inferred that the regulation of thyroid hormone secretion might be closely related to the inhibition of the proliferation of thyroid cancer cells.

## 1. Introduction

Thyroid cancer is one of the most common malignancies of endocrine organs with enormous heterogeneity in terms of morphological features and prognosis [1]. Although most thyroid carcinomas tend to be biologically indolent and have a good prognosis, there are a few associated with more aggressive clinical manifestations [2]. Thus, it is crucial to find effective interventions to slow down or cure the cancer, especially in a personalized manner. It is known that the expression pattern of a gene or a pathway is closely related to the status of a patient and an effective drug could convert the abnormal gene expression in cancer patients to that of the healthy control subjects [3]. For example, the Ras/Raf signaling mechanism plays an important role in thyroid function and many associated genes are abnormally expressed in patients with thyroid cancer [4]. Thus, if a drug has the potential to reverse the gene expression changes and gene regulations of cancer patients to those of the healthy control subjects, this drug might be able to cure the cancer. 

Based on this assumption, the connectivity map (CMAP) project [3] and its continuing project the Library of Integrated Network-based Cellular Signatures (LINCS) [5] have successfully repurposed a few useful drugs and identified several drug targets. Recently, more and more successful applications of CMAP have been reported, such as the study of nematodes to identify compounds that mimic caloric restriction [6,7,8]. However, the CMAP project was conducted on cell lines and it is known that there are substantial differences between cell lines and tissues, which greatly reduces its ability in predicting or repurposing effective drugs. There are also many other computational methods in predicting cancer drugs. For example, in the Cancer Cell Line Encyclopedia (CCLE) project [9], the gene expressions of cancer cell lines were used as features to train an Elastic-Net based drug sensitivity prediction model. However, the model is also based on cell lines and its effectiveness on human tissues is questionable. In addition, only single gene expression variations were modelled in CMAP and CCLE, the gene regulation/co-expression changes or gene network variations were ignored. To this aim, Guney et al. presented a network-based in silico drug efficacy prediction method, which maps drug targets and disease genes into a protein-protein interaction (PPI) network and measures their reachability by the shortest path. However, this method also does not model the network difference between cancer and healthy samples. As a result, a method integrating drug effects on gene expressions at the tissue level and gene network variation between cancer and healthy patients is highly demanded. 

In this paper, we developed a computational pipeline to predict drug response and potential drug target genes on thyroid cancer. Specifically, we first examined several types of perturbations incurred by the development of thyroid cancer comparing to healthy patients, including differential gene expression and differential gene co-expression. After that, we applied weighted gene co-expression network analysis (WGCNA) to construct gene co-expression network for normal samples and cancer samples respectively [10]. WGCNA clusters genes into subnetworks (or modules) by measuring gene-gene expression correlations and their topological overlaps, which are often enriched on specific biological functions. We then searched for drugs and genes whose usage or interference has the potential to reverse the perturbations imposed by thyroid cancer. To achieve this goal, we adopted a pharmacogenomics approach similar to CMAP [3]. Instead of using drug/gene signatures from cell lines, we took the advantage of the signatures derived from tissues [11]. Finally, by combining the top predicted drugs and genes, we inferred potential biological mechanism underlying the inhibition of thyroid cancer cell proliferation.

## 2. Result

### 2.1. Differential Gene Expressions and Their Functions Between Thyroid Carcinoma and Normal Samples

We downloaded the raw count data of 56 normal and 500 thyroid cancer patients from TCGA (https://portal.gdc.cancer.gov/projects/TCGA-THCA) on 21 December 2016. Similar to the Genotype-Tissue Expression (GTEx) project [12], we screened the gene to have at least 0.1 fragments per kilobase of transcript per million fragments mapped (fpkm) in 2 or more individuals and then quantile normalized the gene. 16,575 mRNAs passed the screening and were kept for further analyses. Through differential gene analysis by the software package edgeR [13], we obtained 812 up-regulated differential mRNAs and 213 down-regulated ones. We then performed function analysis of the differentially expressed genes (DEGs) by the David tools (version 6.8) (DAVID; http://david.abcc.ncifcrf.gov/) [14,15]. The up-regulated DEGs are mainly enriched in extracellular matrix (GO:0030198, false discovery rate (FDR) = 7.76 × 10^−7^) and extracellular matrix breakdown (ECM) in terms of biological processes (GO: 0030574, FDR = 2.85 × 10^−5^) and cell adhesion (GO: 0007155, FDR = 4.30 × 10^−7^). In pathway enrichment analysis, the up-regulated DEGs were significantly enriched in neuroactive ligand-receptor interactions (hsa04080, FDR = 7.05 × 10^−5^).

### 2.2. Analysis of Differentially Co-Expressed Gene Pairs between Normal and Diseased Samples

**Figure 1:**
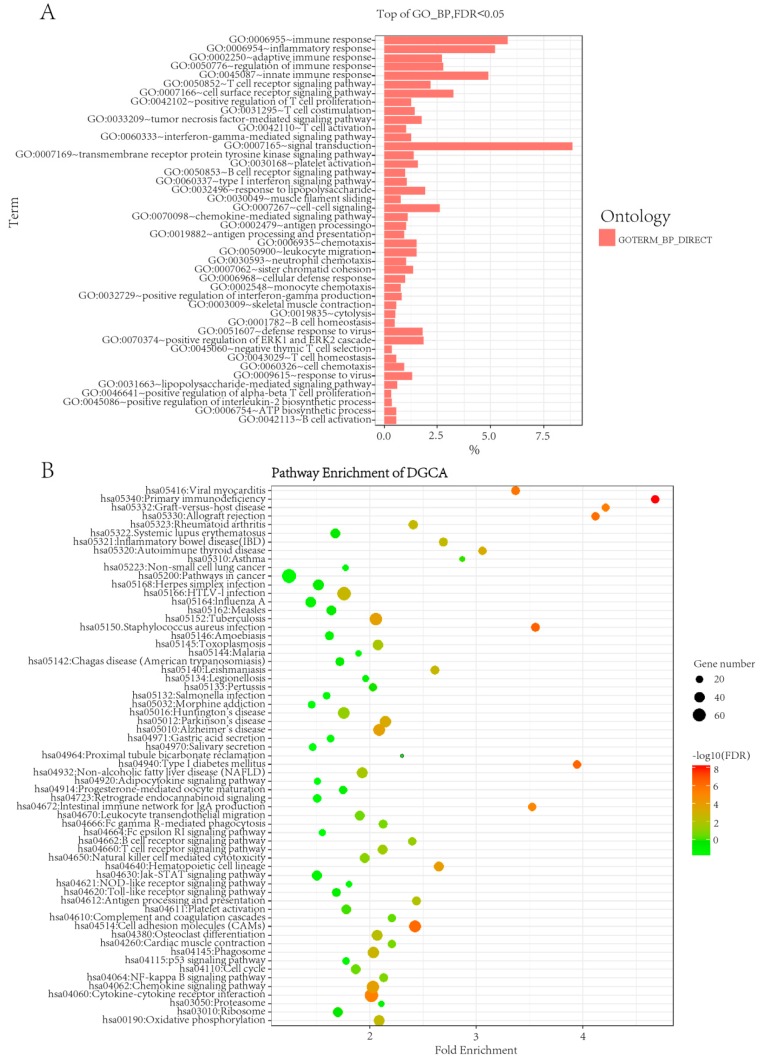
GO and KEGG enrichment of the 2435 unique genes involving in 33,778 significant gene co-expressions identified by DGCA. (**A**) The significantly enriched biological process (GO_BP) with FDR less than or equal to 0.05, where the length of each bar indicates −log10 (FDR) of the corresponding GO BPs; (**B**) KEGG pathway analysis with the row denoting fold change and the size of the dots denoting −log10 (FDR) of the corresponding pathways.

Besides perturbing singe gene expressions, thyroid cancer might also change gene co-expressions. We used an R-package differential gene correlation analysis (DGCA) to identify gene co-expression variations [16]. A total of 33,778 significant gene co-expressions (Dataset S1) were obtained at empirical *p*-value less than or equal to 1.0 × 10^−20^, which involves 2435 unique genes (Dataset S2). We performed function enrichment analysis on Gene Ontology (GO) (see Figure 1A) and Kyoto Encyclopedia Genes and Genomics (KEGG) pathway (see Figure 1B) by the software enrichGO and enrichKEGG. As can be seen from Figure 1A, the 2435 genes were most significantly enriched in GO terms like the “immune response” (GO: 0006955, FDR = 3.34 × 10^−25^) and “inflammatory response” (GO: 0006954, FDR = 6.71 × 10^−22^). Similarly, the top enriched KEGG pathway is “primary immunodeficiency” (hsa05340, FDR = 7.26 × 10^−9^). Our results confirmed the well-known relationship between cancer and immune system and inflammatory responses [17]. 

In addition, we listed in Table 1 the top 10 most significant differential gene co-expressions between normal and cancer samples inferred by DGCA. As can be seen from Table 1 and Dataset S1, most differential co-expressed genes are significantly correlated in normal samples but become less significantly correlated or loose correlation in cancer samples. Specifically, there are 7896 gene pairs significantly correlated in normal samples but have non-significant correlation in cancer samples. In contrast, only 1170 gene pairs show a significant correlation in the diseased sample but no longer have a strong correlation in normal tissues. There are 244 unique genes involved in the 1170 gene pairs and these genes are significantly enriched in muscle activity, such as “myosin slip” (GO: 0030049), FDR = 1.48 × 10^−6^), “muscle contraction” (GO: 0006936, FDR = 1.95 × 10^−7^) and “muscle node tissue” (GO: 0045214, FDR = 1.01 × 10^−4^). 

**Figure 2:**
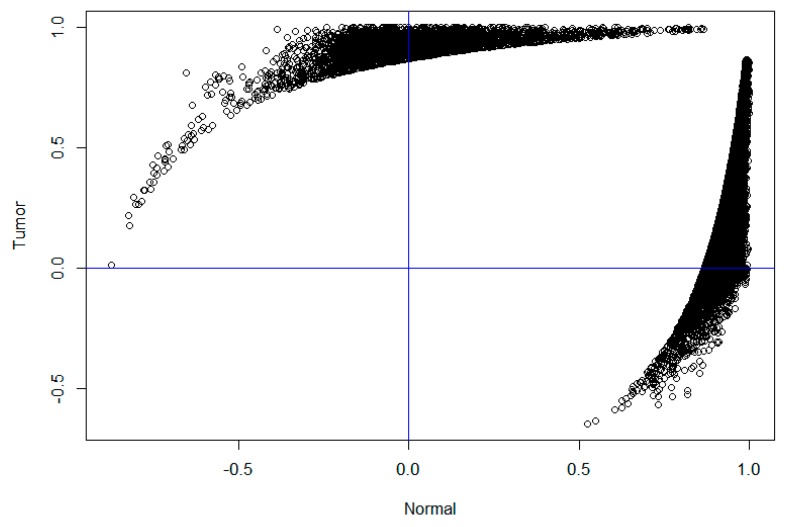
The correlation distribution of differentially co-expressed gene pairs. The X-axis represents the correlation coefficient of the differential co-expressed gene pairs in the healthy samples and the Y-axis represents the correlation coefficient of the differential co-expressed gene pairs in the tumor samples.

For a better view, we plotted the 33,778 significant differential co-expressed gene pairs in Figure 2, in which each node denotes a gene pair, the X-axis denotes their correlation in normal samples and the Y-axis denotes their correlation in cancer samples. It is clear that most nodes are located in the first, second and fourth quadrants. For example, there are 1928 co-expressed gene pairs in the second quadrant, which were negatively correlated in normal samples and positively correlated in cancer samples. The 490 unique genes involved in these gene pairs are significantly enriched in “negative regulation of endopeptidase activity” (GO: 0010951, FDR = 8.37 × 10^−4^), “pre/post mode specification” (GO: 0009952, FDR = 0.01) and “fibrinolysis” (GO: 0044730, FDR = 0.02). Similarly, there are 5090 co-expressed gene pairs in the fourth quadrant, which are positively correlated in normal samples and negatively correlated in diseased samples. The corresponding 1468 genes are significantly enriched in “immune response” (GO: 0006955, FDR = 1.92 × 10^−30^), “adapted immune response” (GO: 0002250, FDR = 1.34 × 10^−18^), “inflammatory response “(GO: 0006954, FDR = 9.31 × 10^−17^) and other immune-related functions. The pathway analysis suggests that these genes are significantly enriched in “cell adhesion molecule (CAM)” (hsa04514, FDR = 2.71× 10^−10^), “the same species Allograft rejection (hsa05330, FDR = 3.90 × 10^−9^) and “primary immunodeficiency” (hsa05340, FDR = 6.23 × 10^−9^). The results suggest that a large network rewiring process has occurred in the normal sample module and many “new” co-expression patterns have emerged, forming a larger module in the diseased sample. The function analysis suggests that these changes may be associated with impaired immune function and low immunity during thyroid cancer.

It is of note that Figure 2 exhibited non-random patterns as a lot of genes have correlations close to 1 in healthy or cancer samples. These might be caused by low expressed genes (with expression level being 0 for most samples), which are prone to technical issues or limitation of sequencing techniques. As a consequence, we might have some false-positive differential co-expressions. Though we have filtered out low expressed genes similar to the GTEx study to reduce the false-positives, it might still not be enough. With the increasing of the sensitivity in RNA sequencing, this issue will be relieved but will not be totally solved. As can be seen, the differential co-expressions and modules we identified have clear biological functions, which might indicate that these analyses still reveal at least partial biological processes involved in the development of thyroid cancer.

We used the weighted gene co-expression network (WGCNA) to construct co-expression network and network modules of healthy and diseased samples respectively. Specifically, we obtained 73 network modules each consisting of highly correlated genes for healthy samples and 53 modules for cancer samples (see Figure S1 for the modules and their relationships). We also listed in Table 2 the detailed information of a few top modules (according to the number of genes in the module). As can be seen, the largest module in the cancer network is the “turquoise” module (hereinafter referred to as the turquoise^tumor^), which consists of 4346 genes. This module is significantly enriched in “transcripts, DNA templates” (GO: 0006351, FDR = 1.31 × 10^−57^; GO: 0006355, FDR = 1.42 × 10^−42^), “protein ubiquitination” (GO: 0016567, FDR = 6.01 × 10^−10^), “Gorky Organization” (GO: 0007030, FDR = 3.01 × 10^−7^) and so on. The second largest module is the “brown” module (referred to as brown^tumor^), which consists of 2550 genes. Interestingly, the module is also significantly enriched in “transcripts, DNA templates” (GO: 0006355, FDR = 9.43 × 10^−6^; GO: 0006351, FDR = 4.34 × 10^−4^). Other large modules in the cancer network are enriched in “mitochondrial translation”, “immune response” and “regulatory regulation of ubiquitin-protein ligase activity involves regulation of mitotic cell cycle transitions”. In contrast, the largest module in the healthy network is the “brown” module (i.e., brown^normal^). The module contains 4469 genes, which are mainly enriched in the “inflammatory response” (GO: 0006954, FDR = 3.98 × 10^−24^). Other modules such as the “blue” module (containing 4240 genes) are mainly enriched in “regulation of GTPase activity” (GO: 0043087, FDR = 7.96 × 10^−5^), the “turquoise” module (containing 3850 genes) mainly enriched in the “translation” (GO: 0006412, FDR = 1.92 × 10^−51^) and the “black” module (containing 3615 genes) mainly enriched in “RNA splicing” (GO: 0008380, FDR = 3.85 × 10^−5^). 

In order to map and compare the global network structure between the normal samples and the diseased samples, we studied the gene overlap among the modules. It was found that the module blue^normal^ and the turquoise^tumor^ are highly overlapped and share 2256 genes. These genes are also mainly enriched in “transcription, DNA template” (GO: 0006351, FDR = 6.44 × 10^−8^) and “protein transport” (GO: 0015031, FDR = 1.26 × 10^−5^). 

### 2.3. Prioritize Drug and Gene Targets Using Online Pharmacogenomics Methods

We next prioritized drugs and drug targets according to their potential to reverse the perturbations imposed by cancer. Specifically, we first queried the CRowd Extracted Expression of Differential Signatures (CREEDS) gene and drug perturbation database [11] using the 812 up-regulated genes and 213 down-regulated genes respectively. The CREEDS database consists of a list of 4295 single drug perturbations and 8620 single gene perturbations obtained using gene expression data collected from GEO. In particular, the CREEDS database is used herein to identify drugs or genetic perturbations that may reverse disease status (i.e., upregulate those inhibiting disease-associated gene expression or downregulate those elevating disease-associated gene expression). The ranking of the drug and gene perturbations is based on the *p*-value of the Fisher’s exact test (see Section 4). We listed the top 10 predicted drugs in Table 3 (left panel). As can be seen, the most commonly predicted drug is baclofen. Though there is no direct indication that baclofen can treat thyroid cancer patients, a couple of studies suggest that baclofen has effects on thyroid hormone levels [18,19]. It can be seen that the top predicted drug (according to *p*-value) is glucocorticoid (GC). GC inhibits the expression of substantially all *IFN-γ* regulated genes, including *IFN-γ* receptor and *STAT-1* and will also block *STAT-1* activation and nuclear translocation. In addition, GC has an anti-apoptotic effect in keratinocytes by inducing anti-apoptosis and inhibiting the expression of pro-apoptotic genes. Moreover, GC has a profound effect on wound healing by inhibiting cell motility and expression of pro-angiogenic factor *VEGF*. 

Though we predicted meaningful drugs by solely using differential genes, theoretically a better drug could reverse other perturbations than gene expression variations (e.g., gene co-expression changes) incurred by thyroid cancer. To this goal, we retrieved the 1958 genes involved in the significant differential gene co-expressions in the second and fourth quadrants in Figure 2, which change the sign of correlation from healthy people to cancer patients. By overlapping the 1958 genes with the 1025 differential genes, we obtained 180 up-regulated genes and 39 down-regulated genes, which were also used to query the CREEDS database. The top 10 predicted drugs were listed in Table 3 (right panel). Interestingly, drugs like baclofen and cidofovir were also ranking in the top, indicating their ability in both reversing single gene perturbation and differential co-expressions. We further predicted drugs according to the 806 differential genes not involving in differential gene co-expressions. The top 20 drugs are listed in the end of this letter. We found that the predicted drugs were all natural chemical small molecules, however they seem not directly relate to thyroid cancer. In contrast, the drug predicted by the 219 differential genes involving in differential gene co-expressions like baclofen and cidofovir are FDA-approved drugs closely related to thyroid cancer. It is clear that the drugs predicted by differential genes involving in differentially co-expressions are more accurate. Studies have found that Cidofovir has potential for the treatment of follicular thyroid cancer [20]. To some extent, inhaled Cidofovir reduces the burden on patients with thyroid cancer [21]. As a result, we could use network information to further refine drugs inferred from single genes.

## 3. Discussion

In this study, we first inferred differential genes between thyroid cancer patients and healthy control subjects. To cross-validate the genes we obtained, we compared them with thyroid cancer associated genes from a few previous studies. For example, Giordano et al. [22] inferred thyroid cancer associated point mutations in *BRAF* and *RAS* genes, which might be driven by the fusion of *RET*, *NTRK1* and *ALK*. Interestingly, *NTRK1* and *ALK* were also inferred to be up-regulated differentially expressed genes in our study, which increase the confidence of our differential genes. we compared the enriched functions of the 219 differential genes involving also in differential co-expressions and those of the 806 differential genes not involving in differential co-expressions. In summary, the 219 differential genes were enriched in GO functions like “cellular protein metabolic” (GO: 0006953) and “acute-phase response” (GO: 0006953) and KEGG pathways like “Primary immunodeficiency” (hsa05340). In contrast, the 806 differential genes not involving in differential co-expressions were enriched in GO terms like “cell adhesion” (GO: 0007155) and “extracellular matrix organization” (GO: 0030198) and KEGG pathways like “Neuroactive ligand-receptor interaction” (hsa04080: Neuroactive). As expected, differential genes involving or not involving in differential co-expressions have different functions. 

By querying the CREEDS database using differentially expressed genes and those involved in differential co-expressions, we prioritized a few drugs for thyroid cancer, such as baclofen, cidofovir, ethanol, glucocorticoid, formaldehyde and 6alpha-methylprednisolone. Previous studies have provided some evidences that these drugs might be indeed related to thyroid cancer and thyroid hormone levels. For example, Baclofen inhibits the release of thyroid stimulating hormone [18]. In addition, the ultrasound-guided ethanol injection (UPEI) treatment is a simple and suitable tool for the treatment of papillary thyroid carcinoma (PTC) cervical lymph-node metastases [23]. Glucocorticoid-induced leucine zippers regulate the proliferation of thyroid cancer cells, which may have potential for cancer treatment [24]. function of Formaldehyde on thyroid may due to a block at the source of thyroid hormone [25]. Finally, methylprednisolone pulse therapy is an effective treatment for thyroid-associated ophthalmopathy with fewer adverse events [26]. It is of note that the drugs provided in the CREEDS database include some molecular compounds and some are still in the early stage of research on cell lines. Further efforts should be put to improve the accuracy of genes associated with drug and gene perturbation. Link prediction paradigms [27] have been applied in the prediction of disease genes [28,29], circular RNA [30], miRNAs [31,32]. Also, computational intelligence [33] such as neural networks [34,35,36] can be applied in the cancer drug field.

It is of note that we adopted a few standard data analysis including edgeR to infer differential genes, DGCA to infer differential co-expressions and WGCNA for network analysis. However, these packages may have their own limitations and the results might slide change if we used other packages for similar purpose. For example, popular differential gene calling software includes limma, edgeR and DESeq2. Limma is mainly used for microarray data and both DESeq2 and edgeR assume the reads follow a negative binomial distribution. DESeq2 tends to call more differential genes than limma and edgeR. Previous studies suggest that the 3 softwares are highly consistent in TCGA studies and thus we believe the main conclusions in this paper maybe independent of the choice of the differential gene calling method.

Finally, we only focused on mRNAs in this study. However, non-coding RNAs also play important roles in the development of thyroid cancer. For example, miRNA-146b [37], miRNA-211 [38] and miRNA-222 [39] are expressed 10 times higher in cancer tissues than in healthy tissues. In addition, the long non-coding RNAs (lncRNAs) associated with thyroid cancer include BANCR [40], FTCSC3 [41] and Ak023948 [42]. However, it is out of the scope of this study.

## 4. Materials and Methods

### 4.1. Gene Co-Expression Network Construction of Normal and Diseased Samples

RNA expression profile data for normal and diseased samples were derived from previous studies [43], which were downloaded from TCGA. In this paper, edgeR was selected to identify the differential expression of RNA from the perspective of universality and validity [13]. The expression values of each gene in the normal sample and the diseased sample were separately quantized to form a standard normal distribution. Then, according to the expression data of normal and diseased samples, the samples are hierarchically clustered, the sample outliers are removed and weighted correlation network analysis (WGCNA) is performed. Finally, the normal sample and the diseased sample network module were constructed by retaining 56 samples from the normal group and 500 samples from the diseased group.

### 4.2. Differential Connection Analysis

DGCA is an R-package used to determine the differential correlation between gene pairs under various conditions [16]. It has many similar features to existing methods of identifying differences. For example, similar to DiffCorr, DGCA converts the correlation coefficient to z-scores and uses the difference in z-scores to calculate the p-value for the differential correlation between genes; however, DGCA divides the differential related gene pairs into nine possible categories. We applied DGCA for differential connectivity analysis. DGCA calculates and analyzes the differential correlation between gene pairs between normal and diseased samples. If its empirical *p*-value (calculated by DGCA) is less than or equal to 1 × 10^−20^, the gene pair is said to have differential connectivity between the two classes of samples. Finally, the degree of enrichment of differential linked gene pairs in each module was evaluated by displacement analysis.

### 4.3. Drugs and Disturbing Gene Labels

4295 drugs and 8620 gene-disturbing tags were collected from the CREEDS database [11]. Enrichment analysis of the perturbed labels of differential genes was performed by Fisher’s exact test. If the drug-induced down-regulated gene overlaps significantly with the up-regulated differential gene, or if the drug-induced up-regulated gene overlaps significantly with the down-regulated differential gene, the drug ranks higher. Single gene perturbations are ranked in the same way. 

## Figures and Tables

**Table 1:** Summary of the top 10 significant differential gene co-expressions identified by DGCA.

Gene1_Sym.	Gene2_Sym.	Normal_cor	Normal_pVal	Tumor_cor	Tumor_pVal	pValDiff
*NOTUM*	*PLD5*	−0.388	0.003	0.992	0	1.40 × 10^−102^
*NKAIN1*	*MATN1*	−0.242	0.068	0.999	0	3.75 × 10^−92^
*MPPED1*	*NKAIN1*	−0.196	0.141	0.998	0	3.62 × 10^−89^
*SOX14*	*PRAP1*	−0.186	0.162	0.993	0	1.44 × 10^−88^
*SOX14*	*MATN1*	−0.171	0.200	0.994	0	1.33 × 10^−87^
*SLC13A5*	*IHH*	−0.167	0.210	0.995	0	2.32 × 10^−87^
*PRAP1*	*IHH*	−0.163	0.220	1.000	0	3.82 × 10^−87^
*DHRS2*	*DPYS*	−0.161	0.228	0.998	0	5.51 × 10^−87^
*GLIS1*	*KCNT1*	−0.157	0.239	0.997	0	9.36 × 10^−87^
*KLHL1*	*SOHLH1*	−0.153	0.250	0.990	0	3.15 × 10^−87^

**Table 2:** The summary of gene number and the main function of the top 5 co-expression models in normal and cancer samples.

Module		Gene No	Function		DEGs_overlap
			Term	FDR	
Normal	Brown	4469	GO:0006954~inflammatory response	3.98 × 10^−24^	237
	Blue	4240	GO:0043087~regulation of GTPase activity	7.96 × 10^−5^	102
	Turquoise	3850	GO:0006412~translation	1.92 × 10^−51^	50
	Black	3615	GO:0008380~RNA splicing	3.85 × 10^−5^	64
	Pink	1190	GO:0006355~regulation of transcription, DNA-templated	2.97 × 10^−106^	4
Tumor	Turquoise	4346	GO:0006351~transcription, DNA-templated	1.31 × 10^−57^	29
	Brown	2550	GO:0006355~regulation of transcription, DNA-templated	9.43 × 10^−6^	29
	Blue	2458	GO:0070125~mitochondrial translational elongation	2.75 × 10^−9^	30
	Thistle2	1973	GO:0006955~immune response	1.78 × 10^−61^	30

**Table 3:** Top 10 drugs predicted by using differential genes and differential co-expressions

1025 Differential Genes		219 Differential Genes Involved in Differential Co-Expressions	
Drug ID	Drug Name	Drug ID	Drug Name
drug:P4898	glucocorticoid|dexamethasone	drug:P5684	cidofovir(2−)
drug:P4171	ethanol|6alpha-methylprednisolone	drug:P5683	cidofovir(2−)
drug:P5683	cidofovir(2−)	drug:P4396	baclofen
drug:P4401	baclofen	drug:P4401	baclofen
drug:P4409	baclofen	drug:P4391	baclofen
drug:P4562	formaldehyde	drug:P4397	baclofen
drug:P4566	formaldehyde	drug:P3977	dimethyl sulfide|dimethyl sulfoxide|solvent
drug:P2096	Erlotinib|dimethyl sulfoxide	drug:P4392	baclofen
drug:P5684	cidofovir(2−)	drug:P3986	dimethyl sulfide|dimethyl sulfoxide|solvent
drug:P4563	formaldehyde	drug:P4438	oxygen atom|2-butoxyethanol

## Supplementary Materials

Supplementary materials can be found at http://www.mdpi.com/1422-0067/20/2/263/s1.

## References

[1] Nikiforov Y.E., Nikiforova M.N. (2011). Molecular genetics and diagnosis of thyroid cancer. Nat. Rev. Endocrinol..

[2] LiVolsi V.A. (2011). Papillary thyroid carcinoma: An update. Mod. Pathol..

[3] Lamb J. (2007). The Connectivity Map: A new tool for biomedical research. Nat. Rev. Cancer.

[4] Salvatore G., De Falco V., Salerno P., Nappi T.C., Pepe S., Troncone G., Carlomagno F., Melillo R.M., Wilhelm S.M., Santoro M. (2006). BRAF is a therapeutic target in aggressive thyroid carcinoma. Clin. Cancer Res..

[5] Duan Q., Flynn C., Niepel M., Hafner M., Muhlich J.L., Fernandez N.F., Rouillard A.D., Tan C.M., Chen E.Y., Golub T.R. (2014). LINCS Canvas Browser: Interactive web app to query, browse and interrogate LINCS L1000 gene expression signatures. Nucl. Acids Res..

[6] Dudley J.T., Sirota M., Shenoy M., Pai R.K., Roedder S., Chiang A.P., Morgan A.A., Sarwal M.M., Pasricha P.J., Butte A.J. (2011). Computational repositioning of the anticonvulsant topiramate for inflammatory bowel disease. Sci. Transl. Med..

[7] Calvert S., Tacutu R., Sharifi S., Teixeira R., Ghosh P., de Magalhaes J.P. (2016). A network pharmacology approach reveals new candidate caloric restriction mimetics in *C. elegans*. Aging Cell.

[8] Mirza N., Sills G.J., Pirmohamed M., Marson A.G. (2017). Identifying new antiepileptic drugs through genomics-based drug repurposing. Hum. Mol. Genet..

[9] Barretina J., Caponigro G., Stransky N., Venkatesan K., Margolin A.A., Kim S., Wilson C.J., Lehar J., Kryukov G.V., Sonkin D. (2012). The Cancer Cell Line Encyclopedia enables predictive modelling of anticancer drug sensitivity. Nature.

[10] Zhang B., Horvath S. (2005). A general framework for weighted gene co-expression network analysis. Stat. Appl. Genet. Mol. Biol..

[11] Wang Z., Monteiro C.D., Jagodnik K.M., Fernandez N.F., Gundersen G.W., Rouillard A.D., Jenkins S.L., Feldmann A.S., Hu K.S., McDermott M.G. (2016). Extraction and analysis of signatures from the Gene Expression Omnibus by the crowd. Nat. Commun..

[12] The GTEx Consortium (2015). The Genotype-Tissue Expression (GTEx) pilot analysis: Multitissue gene regulation in humans. Science.

[13] Robinson M.D., McCarthy D.J., Smyth G.K. (2010). edgeR: A Bioconductor package for differential expression analysis of digital gene expression data. Bioinformatics.

[14] Huang D., Sherman B.T., Lempicki R.A. (2009). Systematic and integrative analysis of large gene lists using DAVID bioinformatics resources. Nat. Protoc..

[15] Huang D., Sherman B.T., Lempicki R.A. (2009). Bioinformatics enrichment tools: Paths toward the comprehensive functional analysis of large gene lists. Nucl. Acids Res..

[16] McKenzie A.T., Katsyv I., Song W.M., Wang M., Zhang B. (2016). DGCA: A comprehensive R package for Differential Gene Correlation Analysis. BMC Syst. Biol..

[17] Adam J.K., Odhav B., Bhoola K.D. (2003). Immune responses in cancer. Pharmacol. Ther..

[18] Elias A.N., Szekeres A.V., Stone S., Weathersbee P., Valenta L.J., Haw T. (1984). Gaba-ergic and dopaminergic regulation of thyroid stimulating hormone. Effects of baclofen and metoclopramide. Horm. Res..

[19] Aoun E.G., Lee M.R., Haass-Koffler C.L., Swift R.M., Addolorato G., Kenna G.A., Leggio L. (2015). Relationship between the thyroid axis and alcohol craving. Alcohol Alcoholism.

[20] Catalani S., Palma F., Battistelli S., Nuvoli B., Galati R., Benedetti S. (2017). Reduced cell viability and apoptosis induction in human thyroid carcinoma and mesothelioma cells exposed to cidofovir. Toxicol. In Vitro.

[21] Pradeep P.V., Jayashree B. (2011). Soap bubble type of calcification in thyroid: A radiological surprise!. Otolaryngol. Head Neck Surg..

[22] Cancer Genome Atlas Research Network (2014). Integrated genomic characterization of papillary thyroid carcinoma. Cell.

[23] Vannucchi G., Covelli D., Perrino M., De Leo S., Fugazzola L. (2014). Ultrasound-guided percutaneous ethanol injection in papillary thyroid cancer metastatic lymph-nodes. Endocrine.

[24] Ayroldi E., Petrillo M.G., Marchetti M.C., Cannarile L., Ronchetti S., Ricci E., Cari L., Avenia N., Moretti S., Puxeddu E. (2018). Long glucocorticoid-induced leucine zipper regulates human thyroid cancer cell proliferation. Cell Death Dis..

[25] Patel K.G., Bhatt H.V., Choudhury A.R. (2003). Alteration in thyroid after formaldehyde (HCHO) treatment in rats. Ind. Health.

[26] Higashiyama T., Nishida Y., Morino K., Ugi S., Nishio Y., Maegawa H., Ohji M. (2015). Use of MRI signal intensity of extraocular muscles to evaluate methylprednisolone pulse therapy in thyroid-associated ophthalmopathy. Jpn. J. Ophthalmol..

[27] Ding J., Guo F. (2017). Identification of drug-target interactions via multiple information integration. Inf. Sci..

[28] Zeng X., Liao Y., Liu Y., Zou Q. (2017). Prediction and Validation of Disease Genes Using HeteSim Scores. IEEE/ACM Trans. Comput. Biol. Bioinform..

[29] Zeng X., Ding N., Rodríguez-Patón A., Zou Q. (2017). Probability-based collaborative filtering model for predicting gene–disease associations. BMC Med. Genom..

[30] Zeng X., Lin W., Guo M., Zou Q. (2017). A comprehensive overview and evaluation of circular RNA detection tools. PLoS Comput. Biol..

[31] Liu Y., Zeng X., He Z., Zou Q. (2017). Inferring MicroRNA-Disease Associations by Random Walk on a Heterogeneous Network with Multiple Data Sources. IEEE/ACM Trans. Comput. Biol. Bioinform..

[32] Zhang X., Zou Q., Rodruguez-Paton A., Zeng X. (2018). Meta-path methods for prioritizing candidate disease miRNAs. IEEE/ACM Trans. Comput. Biol. Bioinform..

[33] Liao Z.J., Li D.P., Wang X.R., Li L.S., Zou Q. (2018). Cancer Diagnosis Through IsomiR Expression with Machine Learning Method. Curr. Bioinform..

[34] Cabarle F.G.C., Adorna H.N., Jiang M., Zeng X. (2017). Spiking Neural P Systems With Scheduled Synapses. IEEE Trans. Nanobiosci..

[35] Song T., Rodríguez-Patón A., Zheng P., Zeng X. (2018). Spiking Neural P Systems with Colored Spikes. IEEE Trans. Cogn. Dev. Syst..

[36] Song T., Zeng X., Zheng P., Jiang M., Rodríguez-Patón A. (2018). A parallel workflow pattern modelling using spiking neural P systems with colored spikes. IEEE Trans. Nanobiosci..

[37] Yip L., Kelly L., Shuai Y., Armstrong M.J., Nikiforov Y.E., Carty S.E., Nikiforova M.N. (2011). MicroRNA signature distinguishes the degree of aggressiveness of papillary thyroid carcinoma. Ann. Surg. Oncol..

[38] He H., Jazdzewski K., Li W., Liyanarachchi S., Nagy R., Volinia S., Calin G.A., Liu C.G., Franssila K., Suster S. (2005). The role of microRNA genes in papillary thyroid carcinoma. Proc. Natl. Acad. Sci. USA.

[39] Pallante P., Visone R., Ferracin M., Ferraro A., Berlingieri M.T., Troncone G., Chiappetta G., Liu C.G., Santoro M., Negrini M. (2006). MicroRNA deregulation in human thyroid papillary carcinomas. Endocr. Relat. Cancer.

[40] Wang Y., Guo Q., Zhao Y., Chen J., Wang S., Hu J., Sun Y. (2014). BRAF-activated long non-coding RNA contributes to cell proliferation and activates autophagy in papillary thyroid carcinoma. Oncol. Lett..

[41] Jendrzejewski J., He H., Radomska H.S., Li W., Tomsic J., Liyanarachchi S., Davuluri R.V., Nagy R., de la Chapelle A. (2012). The polymorphism rs944289 predisposes to papillary thyroid carcinoma through a large intergenic noncoding RNA gene of tumor suppressor type. Proc. Natl. Acad. Sci. USA.

[42] Amaral P.P., Clark M.B., Gascoigne D.K., Dinger M.E., Mattick J.S. (2011). lncRNAdb: A reference database for long noncoding RNAs. Nucl. Acids Res..

[43] Lu M., Xu X., Xi B., Dai Q., Li C., Su L., Zhou X., Tang M., Yao Y., Yang J. (2018). Molecular network-based identification of competing endogenous RNAs in thyroid carcinoma. Genes.

