# Supplementary material for: Molecular Network-Based Drug Prediction in Thyroid Cancer
Source: Int J Mol Sci. 2019 Jan 11;20(2):263. doi: 10.3390/ijms20020263 (PMC6359462; doi:10.3390/ijms20020263)
Supplement: Supplementary file 1 [file ijms-20-00263-s001.zip › Figure S1.pdf]

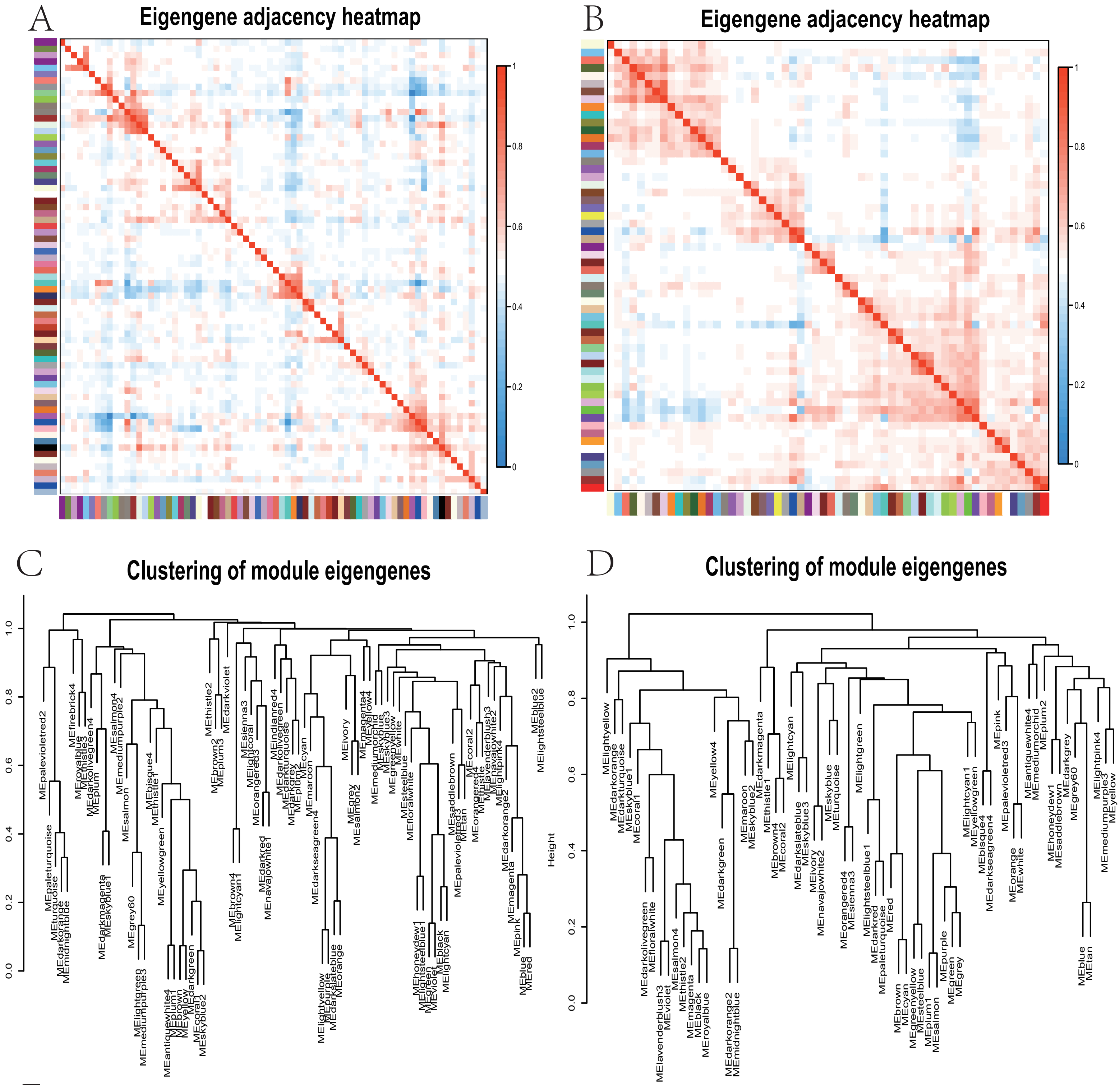

E

Module		Gene number	Function
Normal	Brown	4469	GO:0006954~inflammatory response
	Blue	4240	GO:0043087~regulation of GTPase activity
	Turquoise	3850	GO:0006412~translation
	Black	3615	GO:0008380~RNA splicing
	Pink	1190	GO:0006355~regulation of transcription, DNA-templated
	magenta	906	GO:0045165~cell fate commitment
	greenyellow	650	GO:0007267~cell-cell signaling
	tan	617	GO:0042474~middle ear morphogenesis
	salmon	582	GO:0008584~male gonad development
	cyan	511	GO:1900034~regulation of cellular response to heat
Tumor	Turquoise	4346	GO:0006351~transcription, DNA-templated
	Brown	2550	GO:0006355~regulation of transcription, DNA-templated
	Blue	2458	GO:0070125~mitochondrial translational elongation
	Thistle2	1973	GO:0006955~immune response
	yellow	1854	GO:0006334~nucleosome assembly
	green	1396	GO:0006099~tricarboxylic acid cycle
	red	1343	GO:0006351~transcription, DNA-templated
	pink	863	GO:0007616~long-term memory
	purple	674	GO:0070125~mitochondrial translational elongation
	greenyellow	618	GO:0001525~angiogenesis
